# Enhancing catalytic activity of gold nanoparticles in a standard redox reaction by investigating the impact of AuNPs size, temperature and reductant concentrations

**DOI:** 10.1038/s41598-023-38234-2

**Published:** 2023-07-31

**Authors:** Attia Bano, Asadullah Dawood, Rida, Farhat Saira, Abdul Malik, Musaed Alkholief, Hijaz Ahmad, Muhammad Asad Khan, Zubair Ahmad, Omar Bazighifan

**Affiliations:** 1 Department of Chemistry, National Excellence Institute (University), Islamabad, 04524 Pakistan; 2 Department of Physics, National Excellence Institute (University), Islamabad, 04524 Pakistan; 3 Department of Chemistry, University of Sargodha, Sargodha, 40100 Pakistan; 4 Nanoscience and Technology Division, National Centre of Physics, Shahdara Valley Road, Islamabad, 44000 Pakistan; 5 Department of Pharmaceutics, College of Pharmacy, King Saud University, Riyadh, Saudi Arabia; 6 Near East University, Operational Research Center in Healthcare, Nicosia, 99138, TRNC Mersin 10 Turkey; 7 Department of Computer Science and Mathematics, Lebanese American University, Beirut, Lebanon; 8 Section of Mathematics, International Telematic University Uninettuno, Corso Vittorio Emanuele II, 39, 00186 Rome, Italy; 9 Department of Mathematics and Physics, University of Campania, “Luigi Vanvitelli”, 81100 Caserta, Italy; 10 Department of Mathematics, Faculty of Science, Hadhramout University, 50512 Hadhramout, Yemen

**Keywords:** Chemical engineering, Nanoparticles, Electrochemistry

## Abstract

In this work, the catalytic activity of three different sizes of gold nano particles (AuNPs) (12, 30, and 45 nm) synthesized by the citrate reduction process studied in the conventional redox reaction of K_3_Fe (CN_6_)^−3^ to K_4_Fe (CN_6_)^−4^ using NaBH_4_(reductant) at four different temperatures (5 °C, 10 °C, 15 °C, and 20 °C) and measured by UV–visible spectrophotometry. Comparative kinetic analysis of different sizes of AuNPs including rate constant, activation energy, Entropy values and Frequency of collisions are reported for the first time. Transmission electron microscopy analysis is employed to investigate morphology and particle size. Spherical nanoparticles of size 12, 30, and 45 nm were observed. The UV–visible spectra were recorded at regular intervals, and it was seen that the peak of K_3_Fe (CN_6_)^−3^ decreased gradually with time, at the same time surface plasmon resonance of AuNPs remained constant. As reaction catalysts, AuNPs maintain their optical density which shows their stability during the course of reaction. The kinetic parameters i.e., rate constant, and activation energy (k, t_1/2_, E_a_) were determined for three distinct sizes of gold nanoparticles (AuNPs) using the reductant at various concentrations. The value of k increases by increasing reductant concentration. This rise was significant for the small AuNPs. Increasing gold nanoparticle size (12, 30, 45 nm) decreased rate constant. As the size of AuNPs decreased the E_a_ reduced as well, i.e. 17.325 k cal mol^−1^ for 12 nm, 19 k cal mol^−1^ for 30 nm and 21 k cal mol^−1^ for 45 nm AuNPs. For 50 mM of NaBH_4_, k for 45 nm AuNPs is 0.10728 s^−1^, but for 12 nm AuNPs, the value of k is 0.145 s^−1^, indicating that the 12 nm AuNPs have the greatest rate constant values. The rate of reaction rises with an increase in reductant concentration and temperature, but this increase is significant in the case of small-sized nanoparticles, i.e., 12 nm, which have a high surface area and low E_a_. Besides being a model redox reaction, the reduction of K_3_Fe (CN_6_)^−3^ to K_4_Fe (CN_6_)^−4^ has industrial use in making fertilizers and paint industry, anti-coating agent in colour photography, in dot etching and in amperometric biosensors.

## Introduction

Nanotechnology is one of the most versatile technologies because it can be used to create a wide variety of materials, including nanocomposites^1,2^, bimetallic nanoparticles^1–3^, and quantum dots^4^, all of which have applications as catalysts^5,6^. Emerging therapies such as photothermal cancer therapy^7^, electrochemical sensors^8^, medicinal and health care uses treating COVID-19^9^, and antimicrobial nanoparticles^10,11^ are only a few examples. Large-scale commercial applications in catalysis involving reduction processes based on nanocages, nanoboxes, and nanoparticles^12–18^, in bone malignancies treatment^19^, and nanocarrier creation^20^.

Gold nanoparticles possess peculiar features and have several uses in electronics, photonics, biological sensing, and imaging^21,22^. Physical and chemical characteristics of materials^23,24^ with a high surface-to-volume ratio^25,26^ are influenced by electron mobility. Size and shape-dependent nanocatalysis has emerged as an active research subject^27,28^. In general, when particle size decreased, catalytic activity increased^29^. Gold nanoparticles' catalytic activity is influenced by a number of factors, including their size, concentration, and temperature^30,31^. As catalysis only happens on the surface of metals, an increase in the accessible surface area would considerably improve the catalyst's efficiency, hence reducing its effective cost^32^. Nanoscale metal catalysts have a very high surface area, which provides a substantial quantity of the material to the reactants. In general, particle size reduction boosts catalytic activity. There is, however, a threshold size below which additional reductions inhibit the catalysis^33^. The size and shape of metal nanoparticles have a significant impact on their characteristics^34^. When nanoparticles are used as catalysts, aggregation to generate bulk material and catalyst poisoning are the most common problems. Stabilizers are used to avoid nanoparticle agglomeration. Stabilizing agents are chemical species that prevent nanoparticles from aggregating by forming a double-charge layer on the nanoparticles’ surface. Occasionally, the reducing agents sodium borohydride and sodium citrate also function as stabilizing agents. The partial surface charges produce repulsive interactions between nanoparticles, and sodium citrate may operate as a stabilizer for gold nanoparticles since the negative charge of the ionized carboxyl groups reduces aggregation during catalysis. The citrate ion generates an electrostatic barrier at the gold surface and influences the mass transfer and electron transfer rates across the Helmholtz layer at the gold surface^35^. When a catalyst participates in a chemical reaction, the catalyst is not consumed. However, this does not imply that a particular quantity of catalyst may be used forever. There are a variety of mechanisms that diminish the activity of catalysts. The next sections describe these processes, which may be chemical, mechanical, or thermal in origin. Several consequences, including poisoning, thermal degradation, and fouling, leaching, and chemical deactivation, restrict a catalyst's lifetime^36^. Almost any chemical or molecule that may bind to a catalyst's active site and so block the active sites might be considered a catalyst poison. Due to the fact that these molecules have greater adsorption strengths than other species, the active site becomes blocked. Poisons are determined by the adsorbate’s relative adsorption strength. Mercury, which is often employed in studies of catalyst poisoning, is a well-known catalyst poison^37^. In general, there are two primary techniques based on the order of their synthesis. Specifically, the bottom-up and top-down approaches. In both processes, synthesis may be accomplished in gas, liquid, supercritical fluid, solid, or vacuum states. (a) Particle size, (b) particle shape, (c) size distribution, (d) particle composition, and (e) degree of particle agglomeration^38,39^. In this study, the citrate reduction technique was used. Nanoparticles are characterized in this study using UV–visible spectroscopy and Transmission Electron Microscopy (TEM). TEM is an important characterization technique for directly imaging nanomaterials to acquire quantitative measurements of particle and/or grain size, size distribution, and shape. The Surface Plasmon Resonance (SPR) of gold and silver nanoparticles gives rise to a characteristic absorption peak. The SPR is dependent on particle size and may be utilised to determine the particle size and aggregation state^40^.The red or blue shift of the band may be induced by a change in particle size and the ratio of silver ions to zero-valent silver^41^. Through UV–Vis spectroscopy, the diameter of nano particles may be determined as follows “The ratio of the absorbance of gold nano particles (AuNPs) at the surface plasma resonance peak (A_spr_) to the absorbance at 450 nm (A_450_) is dependent on the particle diameter^42^, and each value of the ratio corresponds to a different diameter. At first, borohydride injects electrons onto gold nanoparticles. The nanoparticles of metal become cathodically polarized and function as electron reservoirs. The process is completed in a following, sluggish phase in which ferricyanide ions diffuse to the nanoparticle surface and are reduced by excess surface electrons^43^. The benefit of utilizing hexacyanoferrate ions for this redox investigation is that iron ions are stable with regard to dissociation in both oxidation states (+ 2 and + 3). The reaction is accompanied by a substantial shift in free energy. Hydrolysis of the borohydride ions may also occur during the reduction of Fe (III) by BH_4_^−^.

1 $$B{{H}_{4}}^{-}+2{H}_{2}O\to 4{H}_{2}+B{O}_{2}$$

No chemical reaction takes place between the reactants and nanoparticles^37^.The gold nanoparticles considerably accelerate the pace of the reaction, but they also alter the order of the reaction with regard to the reactants^30,31,44^. It has been observed that the direct reduction of hexacyano ferrate (III) to hexacyano ferrate (II) using borohydride ions follows zero-order kinetics relative to Fe (CN_6_)^−3^. Consistently, first-order kinetics are found in the presence of gold nanoparticles^11^ It has been observed that when Pt nanoparticles are used, hexacyanoferrate (III) ions react with surface Pt atoms, resulting in Pt dissolution in the form of cyanide complexes and a steady decrease in nanoparticle size. However, in the case of gold nanoparticles, no ferricynide complex formation occurs^32,44^. The significance of the reaction stems from the industrial application of potassium ferrocynide. Potassium ferrocynide is used as a plant fertilizer. It is used as an anti-caking agent in the ink and paint industries. To separate copper from molybdenum ores, as well as in colour photography, etc. In the process known as dot etching, potassium ferricynide is used as an oxidizing agent to remove silver from negatives to positives. In colour photography, potassium ferricynide is used to lower the size of colour dots without diminishing their quantity as a kind of manual colour collection. This chemical is also used in electroplating, dyeing wool, as a laboutaray agent, and as a moderate oxidizing agent in organic chemistry, as well as in black and white photography^35^. As potassium ferricynide is reduced, it is employed in physiology research to increase the redox potential of a solution and may oxidise reduced cytochrome in intact, isolated mitochondria. In addition, it is employed as an electron transfer agent in several amperometric biosensors, substituting oxygen as the natural electron transfer agent in enzymes such as glucose oxidase. In addition to sodium hydroxide and water, it is used to produce Murakami’s etchant.

## Experimentation

The experimental studies to examine the catalytic characteristics of gold nanoparticles for the standard reduction of potassium ferricynide consisted of three stages. Initially, nanoparticles were synthesized using the citrate reduction process, and then they were characterized using UV–visible spectrophotometric and TEM methods. The second stage was to optimize the reaction conditions for potassium ferricynide reduction to potassium ferrocynide employing nanoparticles as a catalyst and sodium borohydride as a reducing agent. Using UV–visible spectroscopy, the last step consisted of investigating the effect of nanoparticle size, temperature, and reducing agent concentration on the reduction process.

### Synthesis

#### Chemicals

The following chemicals are used for the preparation of gold nanoparticles:

ACS reagent tetra chloroauric acid (HAuCI_4_ 3H_2_O), batch # 126K0477, sodium citrate dehydrate 99.0% (m.p = 300 °C, batch # 135K0088), sodium borohydride (m.p = 400 °C, batch # 05,631 TB (product of Rohm and Haas), and potassium hexacyanoferrate (III).

#### Procedure

The citrate reduction process is one of the most widely used techniques for synthesizing AuNPs, which are only stable in solution. This approach was established by Turkevich in 1951 and is generally used for the aqueous synthesis of somewhat polydisperse AuNPs. Ferns studied this process in 1973 and observed that the particle size in the citrate reduction procedure may be altered by adjusting the citrate-to-gold ratio. Trisodium citrate converts Au^+3^ to Au in this process. The citrate ions and oxidation products (such as acetone dicarboxylate) also function as surfactants to bind to the nanoparticles; hence, the AuNPs are stabilized by electrostatic repulsion as the protective ligand. The citrate ion is adsorbed on the gold surface; hence, this approach is often employed to synthesize AuNPs with a loose ligand shell. Gold nanoparticles are produced by reducing auric acid with sodium citrate.

**Figure 1:**
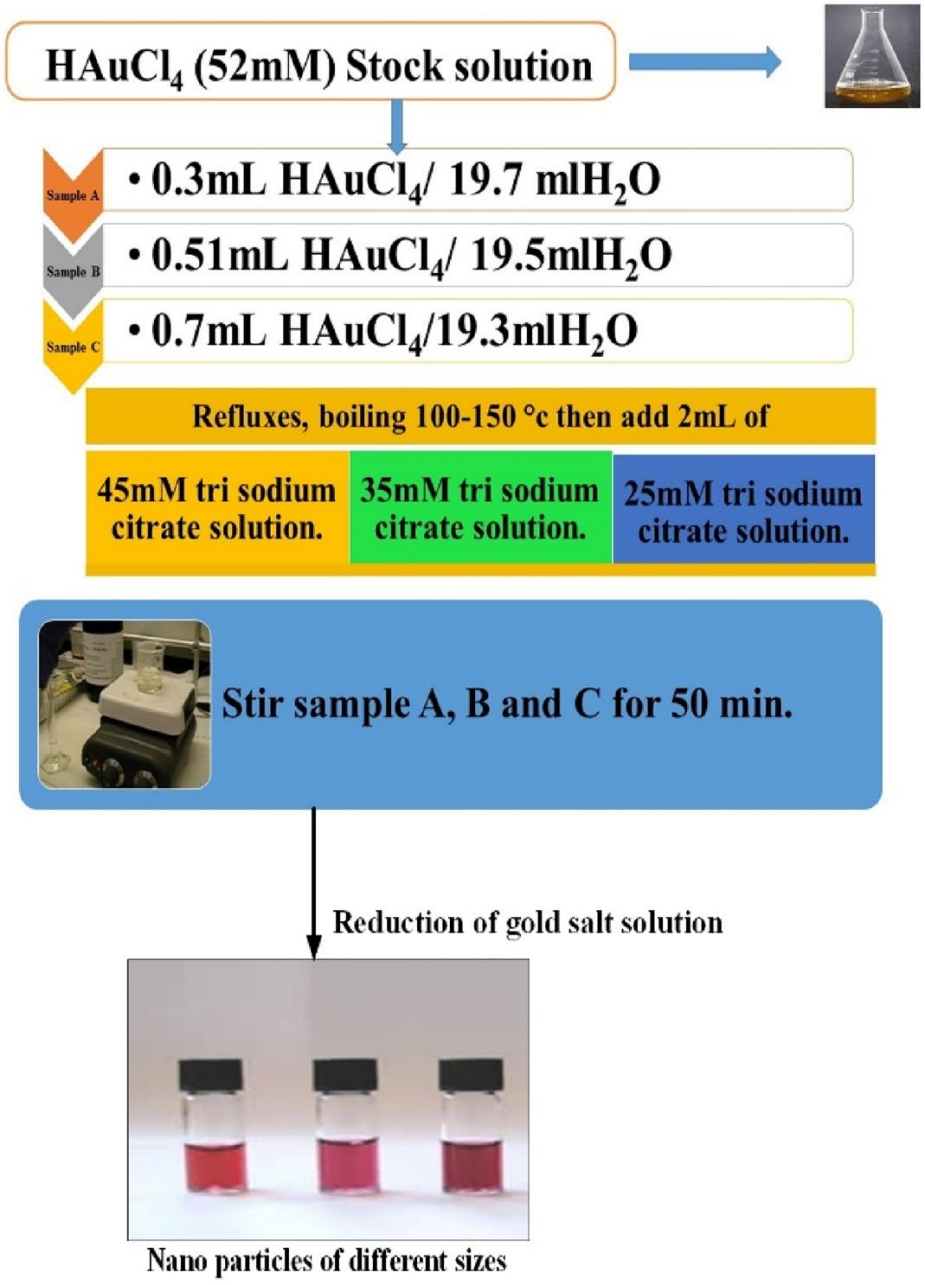
Scheme for synthesis of gold nanoparticles.

The reaction follows a straightforward reduction process in which the HAuCl_4_ solution reduces to metallic gold atoms that combine to form spherical nanoparticles. The reduction process between auric acid and sodium citrate that produces gold nanoparticles is shown in Fig. 1. The method for the synthesis of AuNPs is outlined below.

### Characterization

#### UV analysis

For electronic spectra, a Shimadzu 1601 UV–visible spectrophotometer was used. Using the Shimadzu 1601 UV–visible spectrophotometer with a pair of 1-cm-long quartz cuvettes, the electronic spectra of samples were recorded. To determine the diameter of nanoparticles, UV–visible spectroscopic experiments are carried out. The particle size influences the ratio of the absorbance of AuNPs at the surface plasma resonance peak (Aspr) to the absorbance at 450 nm (A_450_). UV visible spectroscopy was utilised to observe the influence of AuNPs size, reductant concentration, and temperature on the typical redox reaction of potassium ferricynide to potassium ferrocynide. In a typical quartz cuvette, 100 ul of 3 mM potassium ferricynide, 2.5 ml of HPLC water, 100 ul of 3.7 nM AuNPs, and 100 ul of newly synthesized ice-cooled NaBH_4_ were added. To adjust the volume to 3.5 ml, the reductant must be added last since its inclusion initiates the chemical reaction. Due to a time-dependent reaction, the yellow hue of potassium hexacyanoferrate (III) became colourless, and absorbance spectra were recorded at a 1-min time interval (according to the reaction conditions). Each of the four distinct doses of NaBH_4_ (50 mM and 100 mM) and the three different sizes of nanoparticles were repeated in the same experiment. The same experiment was conducted with three different sizes of nanoparticles at four different temperatures of the reaction mixture (5 °C, 10 °C, 15 °C, and 20 °C) to determine the influence of temperature. The Au Nano catalyst was found to be very stable and catalytically effective. Constant concentrations of gold nanoparticles (3.7 nM) and reductant (50 mM) were maintained.

#### TEM analysis

The samples of nanoparticles were quite diluted, thus they were centrifuged to concentrate them. Due to the apparent colors of the particle dispersions, the concentration of the samples was adequate. Before shipment, (Georgia Institute of Technology, Atlanta, Georgia, United States; University of Liverpool, United Kingdom) solutions were put into a compact, leak-proof container. To avoid leakage, the caps were carefully screwed on and sealed with Para film; each sample was properly labelled with its unique ID. Every specimen was encased in a secondary container. A 120 kV FEI Techni Spirit was used to do TEM research on nanoparticles. TEM specimens were made by coating a 400 mesh copper grid with formvar. Under ambient circumstances, a 5 ul drop of diluted nanoparticles was evaporated on the grid, and then samples were photographed using a microscope.

## Results

This study examines the catalytic activity of different sizes of gold nanoparticles in the reduction of potassium ferricynide to potassium ferrocynide, a classic redox process. The catalytic activity is influenced by the size of gold nanoparticles, the temperature, and the concentration of the reductant. In an attempt to clarify these effects, UV/Visible spectrophotometry are used here. The size of nanoparticles was first evaluated using UV–visible spectroscopy and TEM analysis. These nanoparticles were then used as catalysts in a conventional redox process.

### Characterization of gold nanoparticles

**Figure 2:**
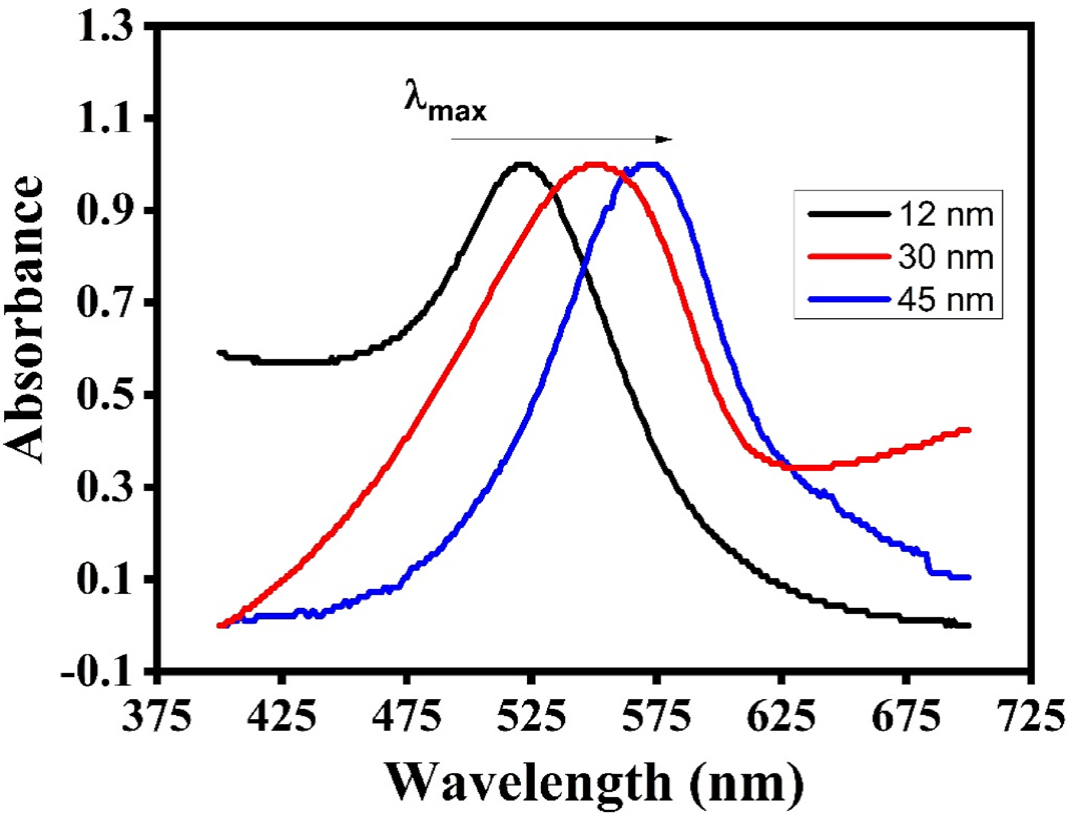
Normalized UV–visible spectra of 12 nm, 30 and 45 nm size.

The citrate reduction procedure was used to produce three distinct samples of gold nanoparticles. The diameter of nanoparticles is then determined using UV–visible spectroscopic techniques as can be seen in Fig. 2. The particle diameter is determined by the ratio of the absorbance of AuNPs at the surface plasma resonance peak (A_spr_) to the absorbance at 450 nm (A_450_). The ratio is as follows:

2 $${A}_{spr}/{A}_{450}=R$$

The ratio of the absorbance to the diameter of the AuNPs as reported in the literature^42^ for three samples is shown in Table 1.

**Table 1:** Absorption ratios and diameters of three samples.

No. of Obs	Sample	A_spr_/A_450_ = R	Diameter (nm)
1	A	0.255/0.163 = 1.564	12
2	B	0.185/0.099 = 1.86	30
3	C	0.137/0.066 = 2.00	45

Thus, particles of size 12, 30, and 45 nm have been synthesized.

### TEM analysis

**Figure 3:**
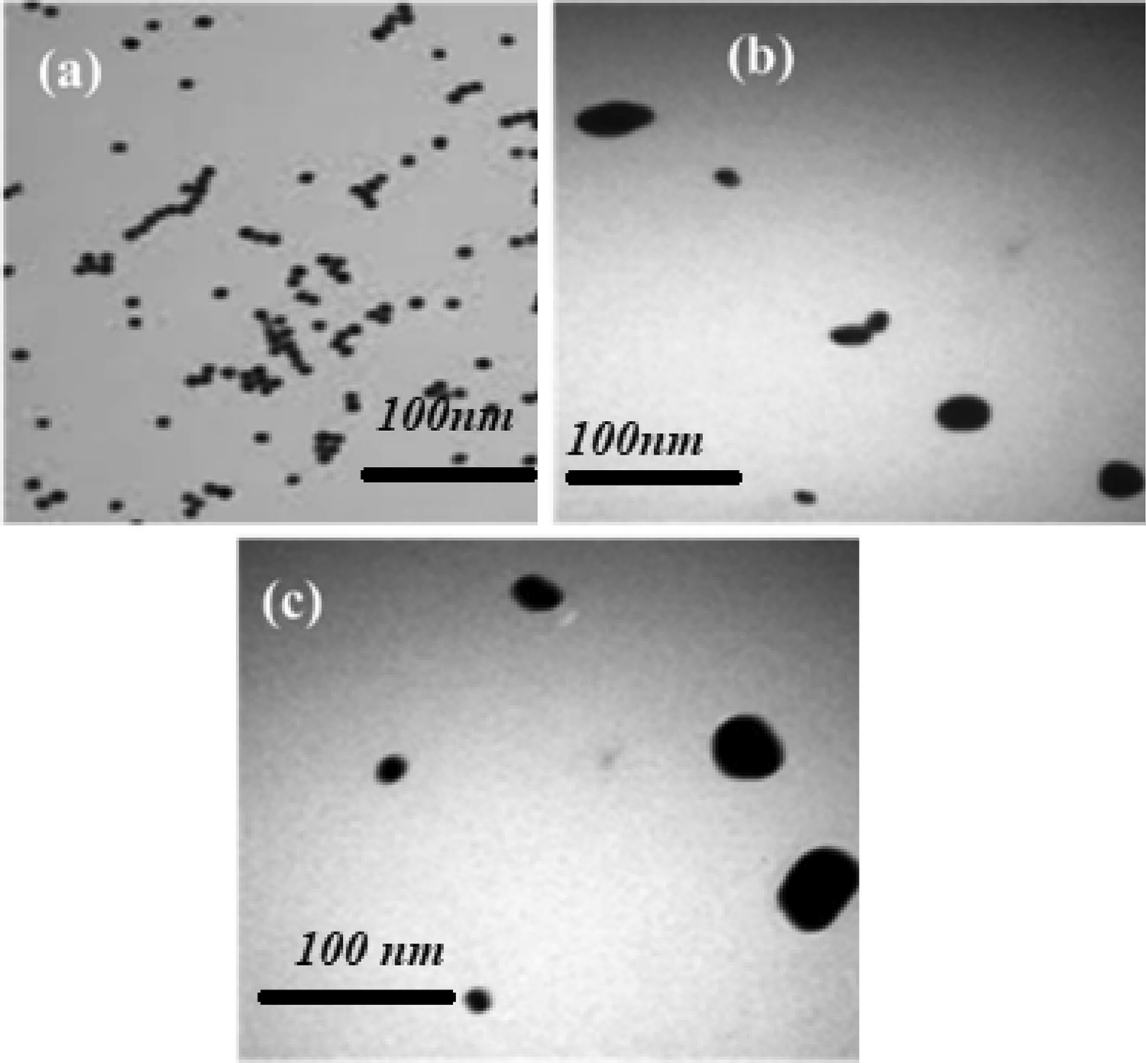
(**a**) A TEM picture of 12 nm gold nanoparticles with a 100 nm scale bar (**b**) TEM picture of 30 nm gold nanoparticles with 100 nm resolution (**c**) TEM picture of 45 nm gold nanoparticles with a 100 nm scale bar.

The homogeneity of three nanoparticle samples was analyzed through TEM. Figure 3a,b,c shows TEM pictures of three distinct sizes of nanoparticles. The TEM pictures of 12 nm (AuNPs) samples are monodisperse, but the images of 30 nm and 45 nm (AuNPs) samples are polydisperse.

### Monitoring the standard redox reaction spectroscopically

Thus, nanoparticles of three sizes, 12, 30, and 45 nm, were prepared and then used as catalysts in standard redox reactions. The standard redox reaction under investigation is as follows:

3 $$\mathrm{B}{{\mathrm{H}}_{4}}^{-}+8\mathrm{Fe}{\left({\mathrm{CN}}_{6}\right)}^{-3}+3{\mathrm{H}}_{2}\mathrm{O}\to {\mathrm{H}}_{2}\mathrm{B}{{\mathrm{O}}_{3}}^{-}+8\mathrm{Fe}{\left({\mathrm{CN}}_{6}\right)}^{-4}+8{\mathrm{H}}^{+}$$

For spectroscopic assessments of catalytic activity in conventional redox processes, pseudo first-order kinetics were used to evaluate the fluctuation of the rate constant with increasing temperature, nanoparticle size, and reductant concentration. The formula is given below

4 $$\ln \left[ {{\text{A}}_{0} - {\text{A}}_{\infty } /{\text{A}}_{{\text{t}}} - {\text{A}}_{\infty } } \right] = - {\text{kt}}$$

In the beginning, the ultraviolet–visible absorption spectra of potassium hexacyanoferrate (III) in water were obtained. There are two absorption peaks at 327 nm and one at 405 nm. Following the addition of NaBH_4_ as a reductant, AuNPs of a constant concentration (3.7 nM) are added.

**Figure 4:**
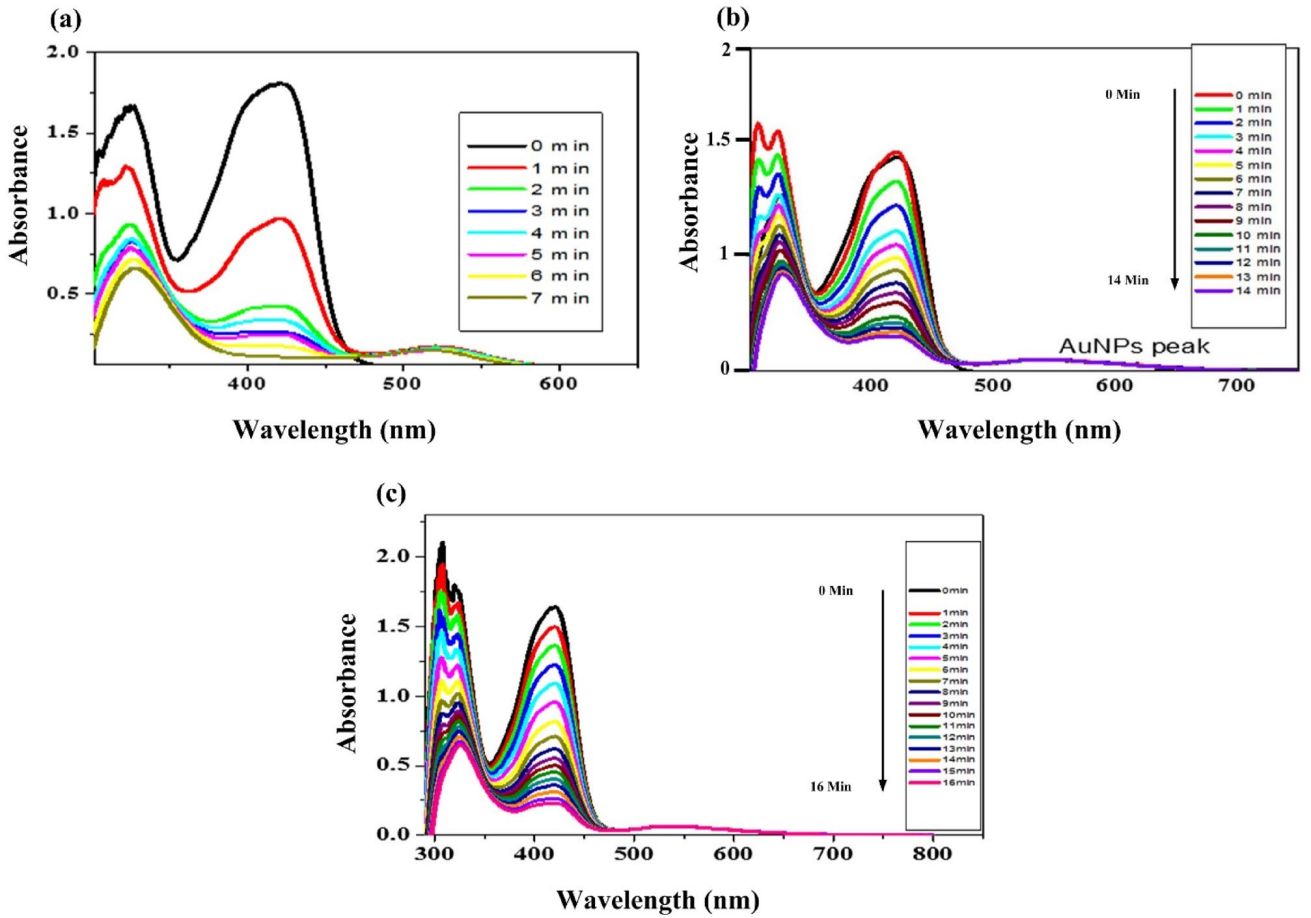
(**a**) 12 nm (**b**) 30 nm (**c**) 45 nm Au nanoparticles on the redox reaction of 30 mM potassium ferricynide at 15 °C Au = 3.7 nM using 50 mM NaBH_4_.

**Figure 5:**
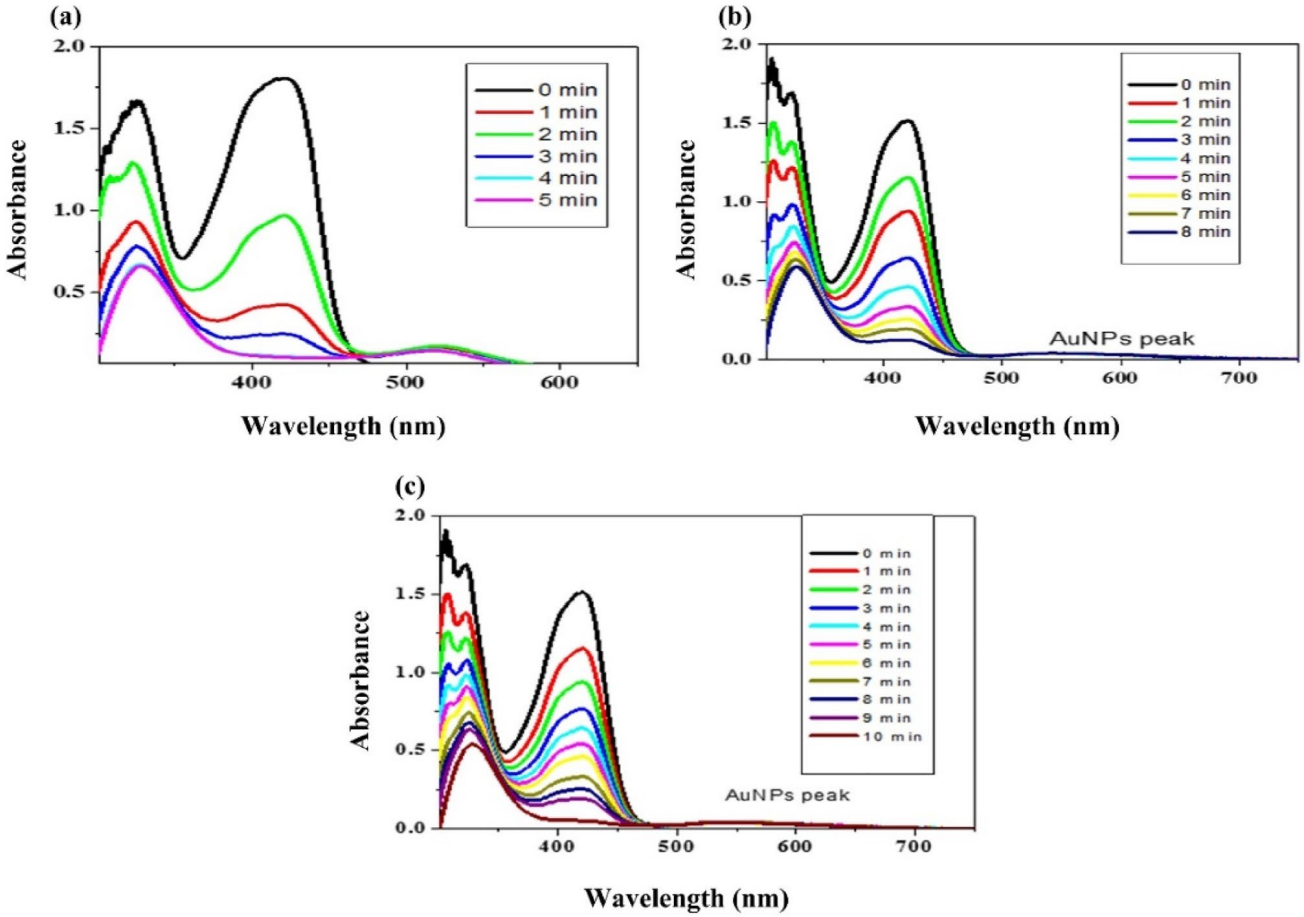
(**a**) 12 nm (**b**) 30 nm (**c**) 45 nm Au nanoparticles resulting from the redox reaction of 30 mM potassium ferricynite at 15 °C and 3.7 nM Au using 100 mM NaBH_4_ Concentration.

**Figure 6:**
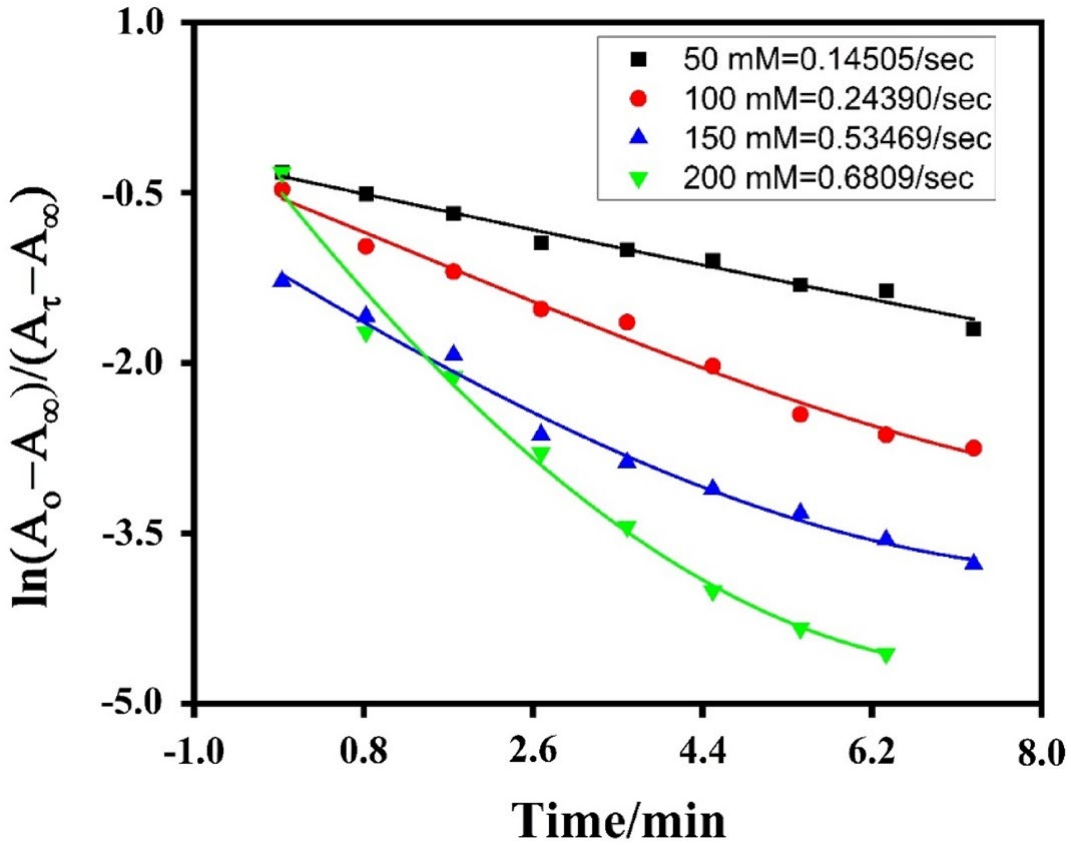
Plot of lnA versus time for 12 nm AuNPs, at 50, 100, 150 and 200 mM NaBH_4_ concentration.

The spectra were then recorded at regular intervals. Peaks of potassium ferricynide were reported to decline gradually over time, while the SPR of AuNPs remained unchanged. This might be explained by the progressive consumption and conversion of ferricynide into potassium ferrocynide. As AuNPs is only a catalyst, the peak of AuNPs has been seen to stay unchanged. Within the specified range, potassium ferrocynide is UV active. Using Eq. (4), the first order rate constant was determined. The rate constant was computed for three distinct AuNPs sizes, four distinct reductant concentrations Fig. 4 (50 mM NaBH_4_ concentration) and Fig. 5 (100 mM NaBH_4_ concentration) and same experiment was done for 150 mM and 200 mM NaBH_4_ concentration to find variation of reductant concentration on reaction rate (Fig. 6).

#### Effect of varying reductant concentration

#### Effect of variation of temperature

**Figure 7:**
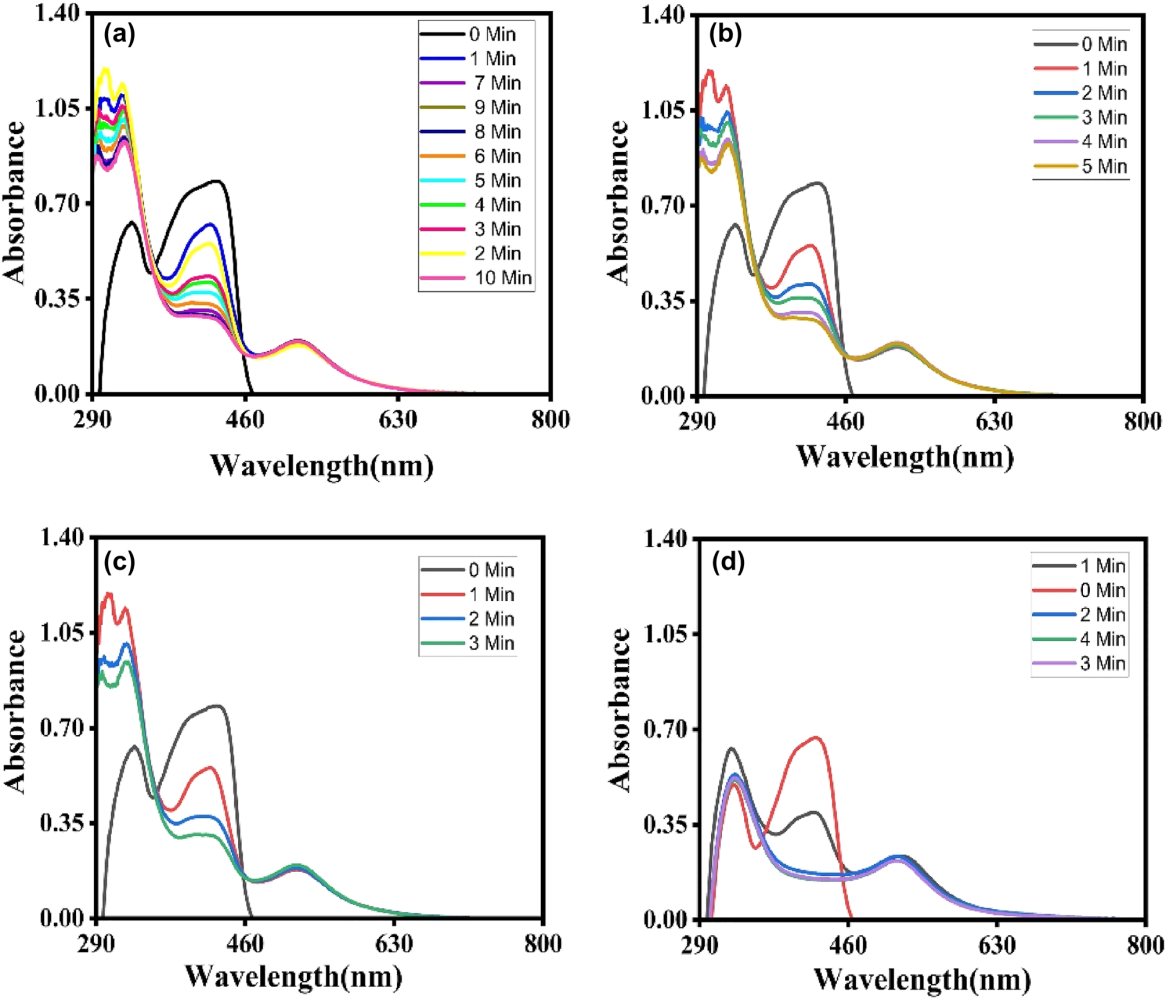
Standard redox reaction of 12 nm Au nanoparticles with 30 mM potassium ferricynide and 50 mM NaBH_4_ Concentration (**a**) at 5 °C (**b**) at 10 °C (**c**) at 15 °C (**d**) at 20 °C.

The impact of change in the reaction mixture on the typical redox reaction was studied spectroscopically by holding the potassium ferricynide and AuNPs concentrations constant and calculating the rate constant using relation 4 at four different temperatures (5, 10, 15, 20 °C), the concentrations of NaBH_4_ and potassium ferricynide were maintained at 50 mM and 30 mM, respectively for three different sizes of AuNPs and for 12 nm AuNPs effect of temperature on reaction rate is shown in Fig. 7

##### Effect of variation of temperature for 12 nm AuNPs

The same experiments for finding the effect of increase of temperature at 5 °C (b) 10 °C (c) 15 °C (d) 20 °C by using (30 nm, 45 nm) AuNPs were also performed and observation was recorded and results are listed in Table 2.

**Table 2:** Results of Temperature effect on standard redox reaction.

No. of Obs	Size of nano-particles (nm)	TEMP (K)	Rate constant/Sec	Activation energy (kcal/mol)	Frequency factor (min^−1^)
1	12	278	0.18863	17.343216	9.90 × 10^12^
2		283	0.38726		
3		288	0.69417		
4		293	0.951		
5	30	278	0.11125	19.023	0.412 × 10^13^
6		283	0.21133		
7		288	0.39932		
8		293	0.66785		
9	45	278	0.08461	21.0156	5.0295 × 10^14^
10		283	0.14166		
11		288	0.293		
12		293	0.46632		

## Discussions

### Spectroscopic measurements of the rate constant by the variation of Concentration of reductant and size of AuNPs

**Figure 8:**
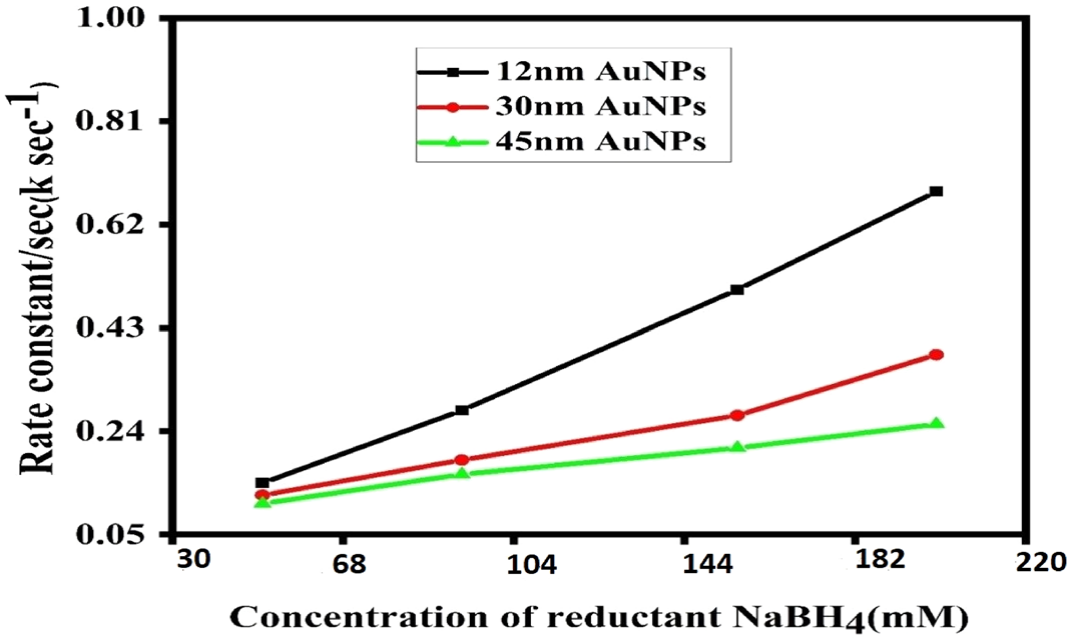
Effect of size of AuNPs and variation of concentration of reductant on standard reaction rate.

The response was observed spectroscopically, by progressively adding NaBH_4_ to the reaction mixture, a time-dependent drop in absorbance was seen by applying different reductant concentration. Equation (4) was used to get the rate constant. The spectra of AuNPs with diameters of 12, 30, and 45 nm and reductant concentrations of 50 and 100 are displayed (Figs. 4, 5). And for the same size of catalyst variation of reductant concentration on the reaction was observed for the reductant concentration at 150 mM and 200 mM NaBH_4_ and summarize the effect for 12 nm AuNPs in (Fig. 8). The spectra were captured at 15 °C with concentrations of 3.7 nM AuNPs and 30 mM potassium ferricynide, respectively. The whole reaction is straightforward to see spectroscopically^32,45,46^. We have observed by increasing concentration of reducing agent rate constant also increases that is highest rate constant for each three sizes is found at 200 mM NaBH_4_ concentration. As shown in (Fig. 6) after reaching to a maximum value, rate of reaction depends on amount of gold nanoparticles. We observe the size of catalyst is the major factor that effect catalytic activity^47,48^. We have summarize the size effect of catalyst on reaction rate with variation of reductant concentration in (Fig. 8).

Using Eq. (4), rate constants are computed and summarized in Table 2. These studies are conducted at a temperature of 15 °C. In discussion of above results we will discuss the following effects on standard redox reaction.

#### Effect of size of AuNPs

Nanoparticles have created a new area of catalysis due to their greater surface-to-volume ratio^27,49^. Electrochemical catalytic characteristics of nanoparticles are sensitive to their size and interface. When nanoparticles are confined to a limited dimension, electromagnetic fields with considerably greater protons and resonance frequencies are generated, resulting in high surface plasmonic characteristics^50^.

Table 2 further reveals that when the size of gold nanoparticles rises, the rate constant drops owing to a reduction in the active surface area of nanoparticles as catalysts^22,51^. For 12 nm gold nanoparticles, the rate constant has the largest value even when the concentration of reductant is low, and in our case, the rate constant were recorded at a reductant concentration of 50 mM (0.14505/s) 100 mM (0.243901/s) 150 mM (0.53469/s) and for 200 mM NaBH_4_ which is (0.243901/s) as shown in Fig. 8.

#### Effect of borohydride concentration

Three different sizes of nanoparticles were used to examine the impact of borohydride concentration. AuNPs were evaluated to be stable over the concentration spectrum. And the rate constant was proportional to the concentration of NaBH_4_ linearly. As the concentration of borohydride rose, a shift in the SPR peak towards the lower wavelength area was detected, which may be related to an increase in the concentration of hydride ions around the catalyst (Fig. 8), resulting in a greater displacement of citrate ions^24^.

Borohydride is capable of injecting electrons into gold nanoparticles, which subsequently function as electron reservoirs and become cathodically polarized^26^. When NaBH_4_ is absorbed on the surface of nanoparticles, H_2_ gas and hydride ions are produced. This blue shift is thus mostly caused by the dielectric change of the surrounding material. When BH injects electrons into gold nanoparticles, citrate ions are removed from their surfaces, resulting in anisotropy of charges on the AuNPs surface and ultimately the aggregation of the catalyst^17,22,52^ using Eq. (4), and the rate constant was determined. By increasing the BH concentration, there is a steady rise, and this increase is greater for tiny AuNPs, i.e., 12 nm, as shown in Fig. 8

#### Arrhenius plots

Arrhenius plots for three distinct AuNPs sizes were constructed. It was observed that the rate constant rose for every 5 °C increase in temperature, with a dramatic increase at 20 °C. In addition, the activation energy was computed by the Arrhenius equation.

5 $$\ln K = \ln A - E_{a} /RT$$

Here, k represents the rate constant, A denotes the frequency factor, R symbolizes the gas constant, T corresponds to the temperature in Kelvin, and Ea represents the activation energy^53^. UV–vis spectroscopic investigation was done with three different sizes of catalyst, and the 12 nm catalyst was determined to have the greatest rate constant at four distinct temperatures. Based on the slope activation energy was estimated for three distinct sizes of AuNPs.

**Figure 9:**
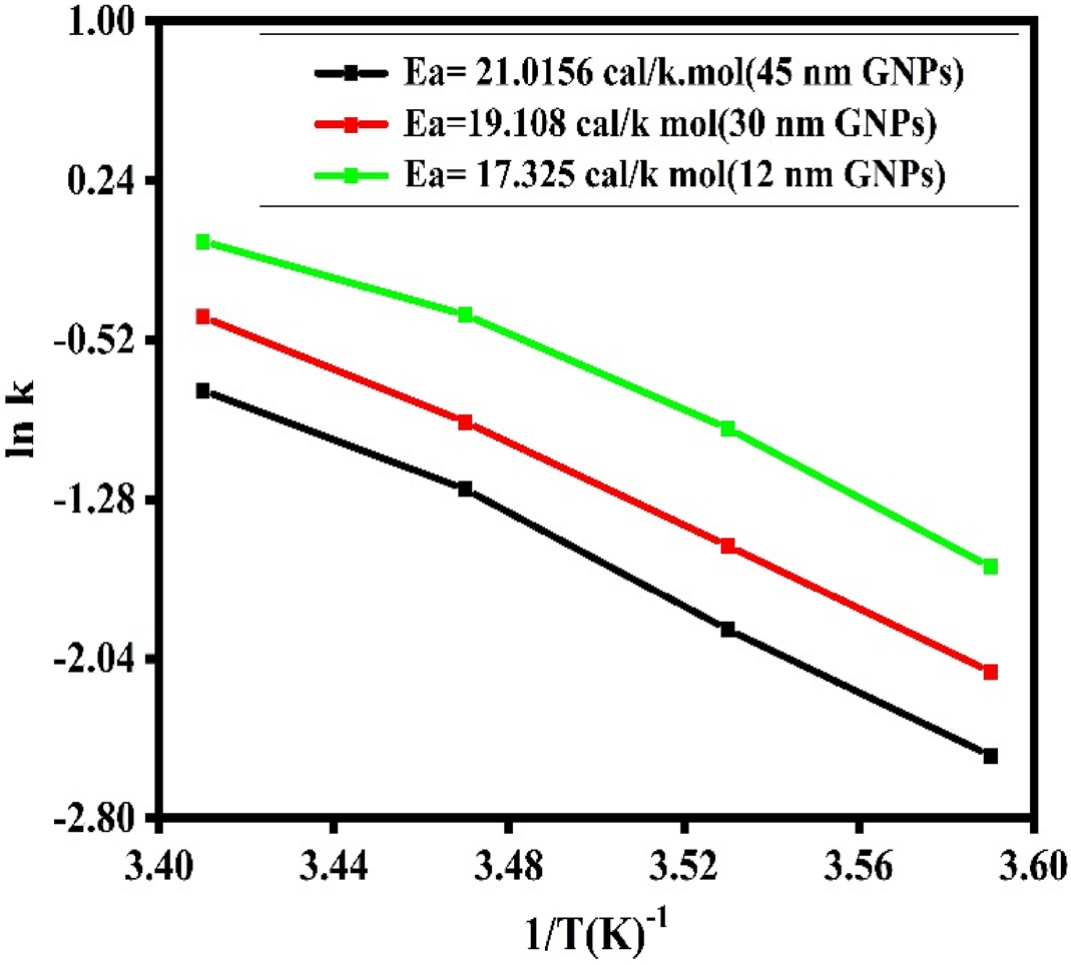
Effect of the temperature on activation energy of different sizes of AuNPs.

Figure 9 demonstrates that when the size of AuNPs lowers, Ea activation energy falls as well; in our study, activation energy drops from 45 to 12 nm AuNPs, with 12 nm AuNPs exhibiting the lowest value. Table 2 contains the values of activation energy and frequency factor.

#### Spectroscopic analysis for measuring rate constant, activation energy, frequency factor

The gold nanoparticles considerably accelerate the pace of the redox reaction, but they also alter the order of the reaction with regard to the reactants. It has been reported that the direct reduction of hexacyanoferrate (III) to hexacyanoferrate (II) with borohydride ions follows zero-order kinetics with regard to [Fe (CN_6_)^−3^] however, first order kinetics have been observed in the presence of gold nanoparticles, as illustrated in Figs. 4, 5 and 6.

Spectrophotometrically, pseudo-first order kinetics, described by Eq. (4), was used, where: ${A}_{\infty }$= Absorbance of potassium ferricynide at infinite time, ${A}_{t}$= absorbance at time t, A_o_ = the absorbance at t = 0 and k = rate constant. From the slope of a straight line, we may compute the rate constant by graphing $\ln \left[ {A_{0} - A_{\infty } } \right]/\left[ {A_{t} - A_{\infty } } \right]$ over time. Generally, the rate of the chemical reaction increases as the temperature rises, and this effect is often represented by the change with temperature of the rate constant k, which follows the Arrhenius equation.

6 $$k = Ae^{{ - Ea/RT}}$$

By calculating the logarithm of each side of Eq. (6), where k = rate constant, A = frequency factor, R = is a constant for all gases, and T = temperature in Kelvin. By graphing lnk vs 1/T, we may get Ea (activation energy) from the slope of the curve (Fig. 9). Consequently, the logarithm of the rate constant is a linear function of the temperature reciprocal. This graph is known as an Arrhenius plot^54^ (Fig. 9). Ea has the same dimensions as R T and is known as activation energy or Arrhenius activation energy. Using these formulae, the computed parameters are listed in Table 2.

Using nanoparticles of varying sizes, the temperature dependency of the reaction is investigated between 5 and 20 °C. All tests with nanoparticles of three distinct sizes are conducted with 50 mM NaBH_4_ and 30 mM potassium ferricynide. All of the solution was thermally stated for a minimum of 5 min inside a stopped-flow spectrophotometer. In this temperature range, AuNPs are continually attacked by solvent and reactant molecules, there is no dissolution, optical density stays constant during the reaction, and the previously reported size and shape of nanoparticles are not altered^25,44^. However, dissolution and instability of catalysts are temperature-sensitive features. Surface atoms dissolve into the reaction mixture at higher temperatures (20 °C). Using Eq. (4) and pseudo-first-order kinetics, the kinetic analysis was performed.

It was observed that when the temperature rises, the rate of reaction increases^55,56^. Using Eq. (6), the activation energy was computed. For small AuNPs, i.e., 12 nm, the maximum rate constant was recorded at four different temperatures, as shown in Table 2. The rate constant fell progressively for 30 nm AuNPs and the lowest rate constant was observed for 45 nm AuNPs. After combining the reactant and AuNPs with borohydride, a maximum of 7 nm of hypso-chromic shift was detected in UV–visible spectra. All of these tests were conducted on a UV–Vis spectrophotometer at 5, 10, 15, and 20 degrees Celsius. In our example, Ea rose as the size of the nano catalyst increased, with values of 17, 19, and 21 kcal/mol for 12, 30, and 45 nm AuNPs, respectively. The increase in rate constant values for smaller nanoparticles corresponds to the higher available surface area for reaction sites and faster diffusion of product formed into the reaction solution. As the AuNPs size increases these available reaction sites decrease in turn decreases the reaction rate. Also, the experiments were conducted at lower temperatures i.e., from 5 till 20 °C to see the impact on this range of temperatures. Our previous published reports^5,6^ on catalysis of nanoparticles for 4-nitrophenol reduction is reported already in which we studied the reaction at higher temperatures i.e., above 20 °C. Current study was dedicated to this lower range of temperatures as catalysis is obviously improved at higher temperatures but our designed catalyst was even useful at lower range of temperatures from 5 till 20 °C. This special characteristic of AuNPs comes from their extra-ordinary reactivity and stability to withstand harsh reaction conditions. Although, AuNPs are somewhat expensive as compared to other alternates like carbon dots, CNTs, graphene and other metal oxides i.e., palladium and platinum but cytotoxicity related with these alternate nanoparticles are reported in literature. While, AuNPs are non-toxic at the same time present excellent performance in high catalytic activities, facile synthesis routes, simple purification, easy recovery, and recyclability, all extensively used in industrial applications.

## Conclusion

We have proven the reduction of ferricynide ion (III) to ferricynide ion (II) by gold nanoparticles of three distinct sizes (at various temperatures and with varied amounts of sodium borohydride). AuNPs function as a very effective catalyst. To validate that AuNPs are actually catalysing reaction a series of control experiments were conducted. Which at the end give qualitative evidence that gold nanoparticles are a superior catalyst compared to gold salt solution and reducing agent used in this experiment. As a catalyst, we observed the experimental condition using three different sizes of nanoparticles. Owing to their high surface-to-volume ratio, high activity, and strong SPR characteristics due to the huge amount of active surface area on the catalyst surface, 12 nm AuNPs resulted in the greatest rate constant. As the size of the catalyst increases, the number of active sites reduces, resulting in a fall in the rate constant. Increasing the size of the nano catalysis also increases the entropy of activation, making the catalyst less selective.

## Data Availability

The data will be available from the corresponding author upon request.
